# Quantum annealing-aided design of an ultrathin-metamaterial optical diode

**DOI:** 10.1186/s40580-024-00425-6

**Published:** 2024-05-09

**Authors:** Seongmin Kim, Su-Jin Park, Seunghyun Moon, Qiushi Zhang, Sanghyo Hwang, Sun-Kyung Kim, Tengfei Luo, Eungkyu Lee

**Affiliations:** 1 Department of Aerospace and Mechanical Engineering, University of Notre Dame, Notre Dame, Indiana, 46556 USA; 2 Department of Applied Physics, Kyung Hee University, Yongin-si, Gyonggi-do 17104 Republic of Korea; 3 Department of Electronic Engineering, Kyung Hee University, Yongin-si, Gyonggi-do 17104 Republic of Korea; 4 National Center for Computational Sciences, Oak Ridge National Laboratory, Oak Ridge, Tennessee, 37830 USA

**Keywords:** Metamaterial, Quantum annealing, Automated design, Active learning, Optical diode

## Abstract

Thin-film optical diodes are important elements for miniaturizing photonic systems. However, the design of optical diodes relies on empirical and heuristic approaches. This poses a significant challenge for identifying optimal structural models of optical diodes at given wavelengths. Here, we leverage a quantum annealing-enhanced active learning scheme to automatically identify optimal designs of 130 nm-thick optical diodes. An optical diode is a stratified volume diffractive film discretized into rectangular pixels, where each pixel is assigned to either a metal or dielectric. The proposed scheme identifies the optimal material states of each pixel, maximizing the quality of optical isolation at given wavelengths. Consequently, we successfully identify optimal structures at three specific wavelengths (600, 800, and 1000 nm). In the best-case scenario, when the forward transmissivity is 85%, the backward transmissivity is 0.1%. Electromagnetic field profiles reveal that the designed diode strongly supports surface plasmons coupled across counterintuitive metal–dielectric pixel arrays. Thereby, it yields the transmission of first-order diffracted light with a high amplitude. In contrast, backward transmission has decoupled surface plasmons that redirect Poynting vectors back to the incident medium, resulting in near attenuation of its transmission. In addition, we experimentally verify the optical isolation function of the optical diode.

**Supplementary Information:**

The online version contains supplementary material available at 10.1186/s40580-024-00425-6.

## Introduction

Photonic devices that have asymmetrical power (intensity) transmission can be considered optical diodes, following the concept of electrical or thermal diodes that have asymmetrical transport properties [1–3]. An optical diode that permits light to transmit in one direction (i.e., forward direction) but blocks it in the reverse direction (i.e., backward direction) has drawn extensive interest. This is because it is needed for various optical applications such as optical information processing [4], photonic integrated circuits [5], and ultrafast pump-probe spectroscopy [6]. The figure-of-merit (*FoM*) of an optical diode can be defined as the difference between the forward (${T}_{F}$) and backward (${T}_{B}$) transmissivities (i.e., *FoM*$={T}_{F}-{T}_{B}$). A familiar example of the optical diode is a macro-scale Faraday rotator, which is commercially available with an *FoM* of 0.7–0.9 at visible and near-infrared spectrums [7]. It exhibits an optical isolation function with a strong magnet or sufficiently long length (e.g., millimeter scale) of a rotating medium [7]. This has been used successfully for high-power laser applications. However, Faraday rotators are generally excessively large for miniaturized photonic circuits owing to their macroscopic size.

**Fig. 1:**
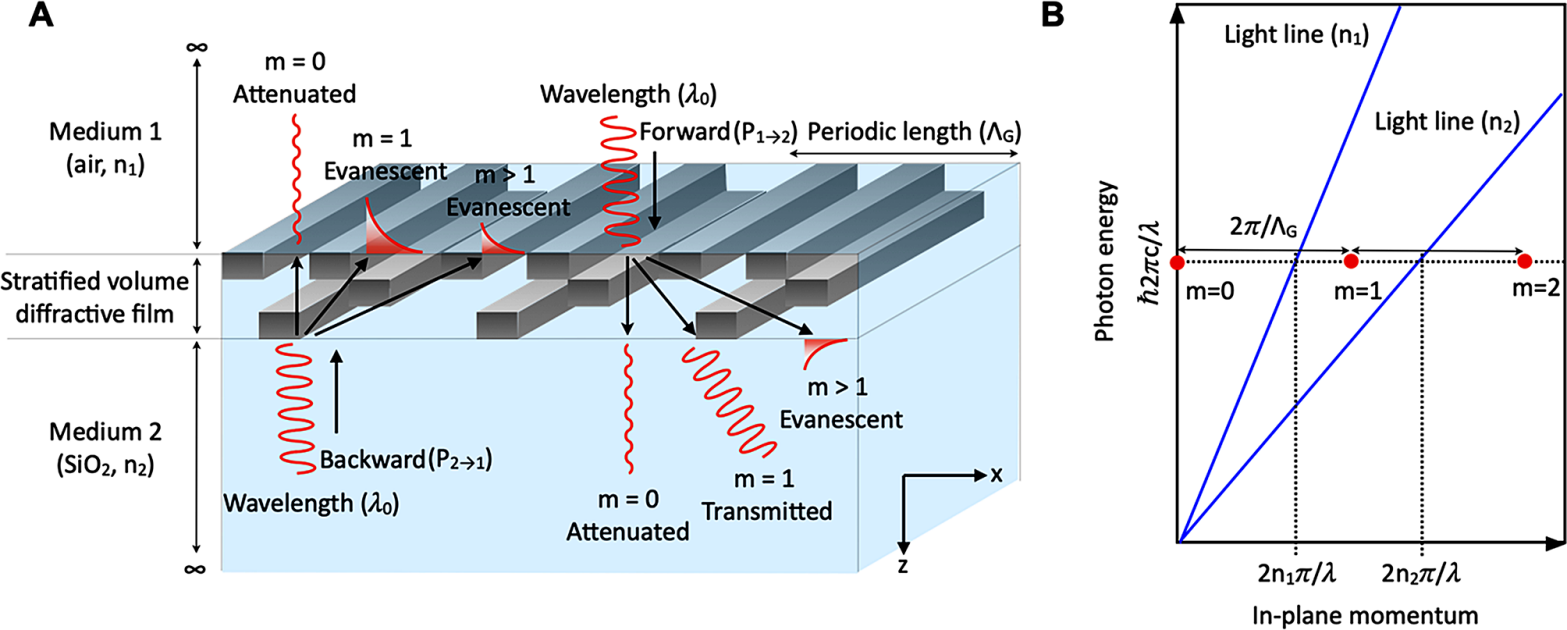
Schematic of a thin-film optical diode with a stratified volume diffractive film (**A**) Optical configuration of a thin-film optical diode consisting of medium 1, stratified volume diffractive film, and medium 2. “Forward” (or “Backward”) indicates that the light with a wavelength of ${\lambda }_{0}$ is normally incident toward the diode from medium 1 to medium 2 (or from medium 2 to medium 1). The refractive index of medium 1 (or medium 2) is n_1_ (or n_2_), and n_2_ is higher than n_1_. *x* and *z* denote the directions of the Cartesian coordinates. The red solid lines indicate electromagnetic waves, and “m” depicts the order of diffracted or evanescent light. The black arrows schematically indicate the direction of k-vectors of diffracted or evanescent lights. (**B**) Schematic illustrating the dispersion relationship between photon energy and in-plane momentum. The blue solid lines indicate the light lines of media 1 and 2. The red dots denote the in-plane momentum of the zeroth-order (*m* = 0), first-order (*m* = 1), and second-order (*m* = 2) diffracted lights. The horizontal black arrow indicates the magnitude of grating momentum (${2\pi /{\Lambda }}_{G}$)

The recent increase in interest has focused on utilizing stratified volume diffractive films composed of metal and dielectric materials (as shown in the example in Fig. 1A) [8–15]. This has unlocked the potential for optical isolation across the thin-film structure. Previous studies have employed various approaches including plasmonic dichroism [8, 9], enhanced transmission through a subwavelength metallic slit [10], tapered metallic gratings [11], and coupled complementary metallic gratings [12, 13]. These investigations yielded effective structural models characterized by representative geometrical parameters. For instance, Xu et al. [8] proposed a model involving two Ag gratings with distinct grating constants separated by a 200 nm-thick SiO_2_ spacer. Under this configuration, light can be transmitted exclusively when the surface plasmon polaritons (SPPs) of the two gratings are coupled. This results in an *FoM* of 0.82 at the target wavelength of 921 nm. Battal et al. [10] identified that the extraordinary transmission through a 210 nm-wide nanoslit exhibits robust unidirectional light transmission when coupled with the SPPs of dual Au gratings. It shows a *FoM* of 0.17 at the target wavelength of 1,560 nm. Li et al. [12] introduced a concept that included coupled complementary subwavelength Ag gratings consisting of an upper grating, a 200 nm-thick SiO_2_ spacer, and a lower grating. The optical isolation function can be observed when the lower grating is embedded within the SiO_2_ medium while the upper grating is in the air. It demonstrates an *FoM* of 0.83 at the target wavelength of 1,250 nm.

Although previous studies have demonstrated potential results, identifying an appropriate structural model for a desired wavelength within the same or adjacent electromagnetic bands remains challenging and complex. This is because the geometries of stratified volume diffractive films can be arbitrary without specific rules of thumb or preferred guidelines. Consequently, the design space for these films can become extensive owing to their wide array of potential geometries. Therefore, a potentially effective strategy involves transforming a continuous and extensive parametric space into a discrete and countable domain [16–18]. Within this discretized parametric space, the resolution, which relates to the nominal distance between two adjacent points, should be high to effectively describe the original continuous parametric space (i.e., the nominal distance should be short). However, locating an optimal state (i.e., an optimal structural model) in such high-dimensional discretized problems is still challenging [19, 20]. Although several algorithms have been proposed to optimize optical materials, optimizing optical diodes having high-dimensional design spaces is still challenging [16, 21–23]. This is because the computational costs (e.g., memory or time) of discretized optimization algorithms based on classical computers generally scale up exponentially or follow a power law with the size of the discretized parametric space.

Quantum annealing (QA) provides a solution to the challenge of scaling-up computational costs by harnessing the natural evolution of a physical system toward its lowest energy state [18]. This process facilitates the identification of optimal states in discretized optimization problems. The key advantage of QA is that it can efficiently evaluate potential solution spaces for complex binary optimization problems regardless of the number of Hamiltonian eigenstates, due to its utilization of coupled and coherent superconducting qubits [24–28]. However, contemporary QA backends such as D-wave 2000q or Advantage have limitations [29, 30]. These are designed to accept only a specific format of eigenstates and the corresponding objective functions defined by the Ising model or quadratic unconstrained binary optimization (QUBO) problem [31, 32]. Additionally, the maximum binary vector length is limited to approximately ∽120 when addressing fully-connected-qubits topologies [30]. Consequently, the current scope of QA in photonics remains confined to a narrow range of applications such as the design of a transparent radiative cooler [17, 33], thermal emitters [34, 35], and a solar absorber [36]. Accordingly, there is a significant need to expand the boundaries of QA applications.

In this study, we proposed an efficient design scheme for a thin-film optical diode consisting of a stratified volume diffractive film aided by QA and active learning. The stratified volume diffractive film was discretized into rectangular pixels, where each pixel was assigned to either a metal or dielectric material and thus, could be represented as a binary vector. A surrogate model formulated using supervised machine learning (e.g., factorization machine (FM)) was used to map the design into a binary optimization problem (e.g., QUBO) compatible with QA. The design scheme was used to automatically identify the optimal material state of each pixel at a given wavelength in the visible–near-infrared region. As a result, the QA-enhanced active scheme with the maximum 40-bit length can optimize 130 nm-thick optical diodes, thereby exhibiting a strong optical isolation function with an *FoM* of 0.81–0.86 at the wavelengths of 600 nm, 800 nm, or 1000 nm. Moreover, we selected an optical diode structure considering the convenience of fabrication (a smaller number of physical layers) from a cluster of good candidates and experimentally validated the optical isolation function of the designed thin-film optical diode.

## Methods

### Fabrication of optical diodes

Considering the convenience of nanofabrication and high *FoM*, we fabricated an *N* = 16 optical diode for ${{\uplambda }}_{0}$ = 800 nm using e-beam lithography (EBL; EBPG5200, Vistec), an e-beam evaporator (EBE; FC-1800, Temescal), and plasma-enhanced chemical vapor deposition (PECVD; 790 series, Unaxis). For EBL, a 330 nm-thick double-layered e-beam resist (first layer: 150 nm of PMMA 950 C2; second layer: 180 nm of MMA EL6, MicroChem) was spin-coated on a UV-fused silica (MES Supplies) substrate. Then, 10 nm of Cr was deposited on the e-beam resist using EBE to measure the distance between the sample surface and e-beam. The thin Cr layer was also used to dissipate accumulated charges during the EBL process [4]. The desired patterns were drawn by the EBL with a 0.5 nm electron beam. The UV-exposed resist was dissolved by a developer solution (isopropanol: deionized water = 3:1) for 50 s. Ti (3 nm) was deposited using the EBE before depositing Ag to increase the adhesion between Ag and SiO_2_. Metal pixels were obtained on the SiO_2_ substrate by dissolving the unexposed resist with a lift-off process using acetone (VWR Chemicals) for 3 h. Dielectric pixels were obtained by depositing SiO_2_ using plasma-enhanced chemical vapor deposition (PECVD). Subsequently, a layer of the metamaterial structure was prepared. Multilayered optical diodes can be fabricated by repeating this process several times.

### Measurement of optical diode performance and morphology

The transmissivities of the fabricated optical diode were measured using a customized optical microscope combined with motorized stages. A linear polarized Gaussian beam from a supercontinuum laser (SuperK COMPACT, NKT Photonics) passing through an objective lens in the optical microscope was loosely focused on the optical diode, mimicking the TM-polarized normal-incident plane wave. The beam spot diameter was ∽ 10 μm using a 4f system, iris, and an objective lens (10×, WD:33, Edmund Optics). A spectrometer (Maya2000 Pro, Ocean Optics) was used to measure the light intensity after transmission through the optical diode. The transmissivities in the forward and backward cases were measured by scanning the entire surface area of an optical diode. During the scanning, an in-house XY translator (step size: 25 μm) made the sample slide while fixing the focus of the beam. The transmissivity for the forward case was measured by simulating the light transmission with a hemicylindrical prism as an index-matching gel and an integrating sphere to capture the diffracted light. The measured transmissivity (${T}_{ }$) was derived using the equation ${T}_{ }= \frac{{T}_{sample}}{{T}_{glass}}$. In this equation, ${T}_{sample}$ represents the relative transmissivity of the measured point, and ${T}_{glass}$ represents the relative transmissivity of the UV-fused silica. These values were determined after light passed through the optical diode. Magnified images were acquired using an optical microscope (Oxford, UK) to observe the optical diode surface. The cross-sectional area of the optical diode was observed via transmission electron microscopy (TEM) using a field-emission scanning electron microscope (S-4800, Hitachi).

### Simulation

The finite element method (COMSOL Multiphysics) was used to solve Maxwell’s equations to obtain the distribution of electromagnetic fields. We set the height of the upper and lower (air and SiO_2_) media (in the z-direction) as 1,600 nm, and the width of the media (in the x-direction) as the periodic length (${{\Lambda }}_{G}$ = 450, 600, and 750 nm for target wavelengths ${{\uplambda }}_{0}$ = 600, 800, and 1000 nm, respectively). Then, we configured structures of the optical diodes following the optimization results (pixel thickness: 20 nm, and width: $\frac{4{\Lambda }}{{N}_{ }}$nm where $N$is the number of pixels). Floquet periodic boundary condition was applied, and finer meshes were applied near pixelated structures. The input light source had a magnetic mode field amplitude in the y-axis to realize the TM-polarized incident light, and the incident angle was set to 0. The field maps were obtained after normalizing the electric field (z-component), and the black arrow showed the Poynting vector at the given spatial point (length was on a logarithmic scale).

## Results and discussion

### Design of optical diodes

Let us consider an optical configuration consisting of medium 1 (air), a stratified volume diffractive film, and medium 2 (SiO_2_). Here, the film exhibits periodicity along the interface, similar to a thin-volume grating (Fig. 1A), and the refractive index of medium 2 (n_2_) is higher than that of medium 1 (n_1_). In this context, the Floquet condition ensures that incident light acquires photon momentum quantized by $$\raisebox{1ex}{$2m\pi $}\!\left/ \!\raisebox{-1ex}{${{\Lambda }}_{G}$}\right.$$ where $${{\Lambda }}_{G}$$ is the periodic length of the diffractive film, and $$m$$ is an integer ($$m$$ = 0, ± 1, ±2, …) [32]. This interplay can result in a robust optical isolation function when the following three conditions align: (1) $$\frac{{2\text{n}}_{1}\pi }{{\lambda }_{0}} <\frac{2\pi }{{{\Lambda }}_{G}}<\frac{2{\text{n}}_{2}\pi }{{\lambda }_{0}}$$ is satisfied (see Fig. 1B), where $${\lambda }_{0}$$ is the wavelength of the incident light, and $$\frac{2\pi }{{{\Lambda }}_{G}}<\frac{2\text{m}\pi }{{\lambda }_{0}}$$ for m > 1; (2) complete attenuation of the zeroth-order diffracted light (m = 0); and (3) amplification of the first-order diffracted light as high as possible. Under these prerequisites, when light strikes the system from medium 1 (the “Forward” case in Fig. 1A, which is denoted as $${\varvec{P}}_{1\to 2}$$), only the first-order diffracted light retains the capability to propagate into medium 2. In contrast, when light travels from medium 2 (the “Backward” case in Fig. 1A, which is denoted as $${\varvec{P}}_{2\to 1}$$), the first-order diffractive light transitions into an evanescent mode within medium 1, effectively blocking light transmission. However, identifying a structural model that satisfies the second and third conditions for a specific wavelength in the desired band (e.g., visible or near-infrared) is challenging. The optimal design of the structure aims to maximize the $${T}_{F}$$ while minimizing $${T}_{B}$$, thereby achieving a high *FoM*.

**Fig. 2:**
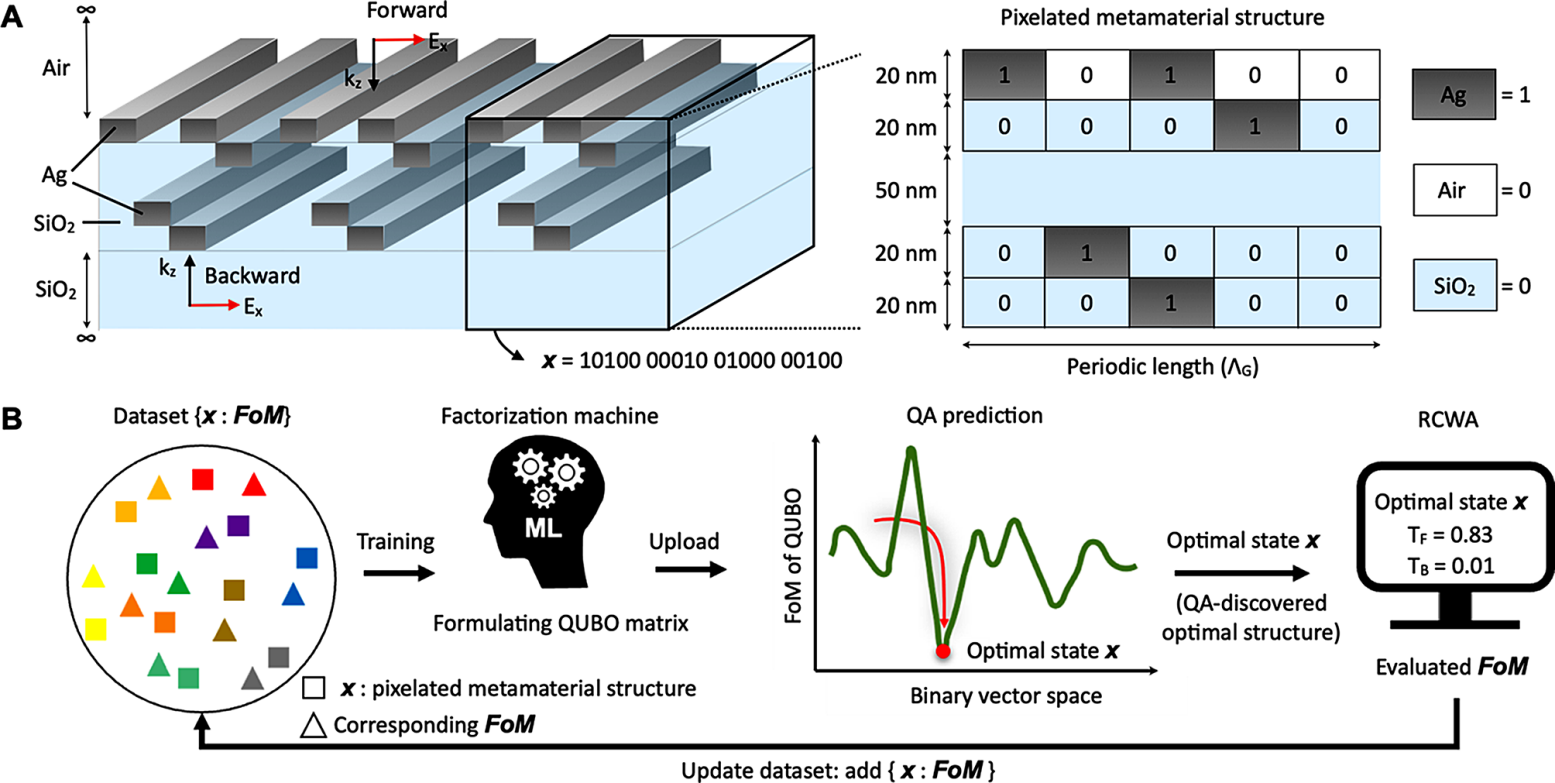
Schematic of a pixelated metamaterial structure with the QA-enhanced active learning scheme. (**A**) Schematic structure of a stratified volume diffractive film used in this study. The cuboid highlighted by black solid lines is a unit cell, which is represented as a pixelated metamaterial structure. E_x_ and k_z_ denote the electric field and the wave vector of the normal-incident light, respectively. (**B**) Diagram of the iterative QA-enhanced active learning scheme. The dataset consists of binary vectors ($\varvec{x}$) and their associated *FoMs*. The factorization machine trained by the dataset formulates the QUBO matrix. The QA identifies the optimal binary vector ($\varvec{x}$) based on the QUBO matrix. The *FoM* of the optimal binary vector is evaluated with RCWA. The optimal binary vector and the *FoM* are added to the dataset for the next optimization cycle

To address the inherent complexity, a stratified volume diffractive film can be discretized into a set of finite-size pixels (i.e., “a pixelated metamaterial structure”). This structure forms a periodic array of unit cells, where each unit cell is discretized into *N* rectangular pixels (Fig. 2A). The unit cell consists of five layers with a 50 nm-thick SiO_2_ spacer as a middle layer. It is noted that the dielectric spacer has demonstrated its capacity to enhance the optical isolation function by facilitating an improved coupling of SPPs within the stratified volume diffractive film [8–15]. The remaining four layers are pixelated. Each pixel can be either a dielectric (“air” for the top layer or “SiO_2_” for the other layers) or metallic (Ag) medium. Here, these materials can support surface plasmons at the Ag/SiO_2_ interface in the visible–near-infrared wavelength range [12]. The material selection of a pixel can be encoded into a binary digit as “0” for a dielectric or “1” for a metal medium. The pixelated metamaterial structure is then represented by a binary vector (***x***) with a length of *N*: {${x}_{i}|{ x}_{i}\in$[0,1], *i* = 1, 2, … *N*}. An *N*-pixel unit cell has 2^*N*^ possible configurations, and a unit cell with finer pixels can potentially yield a better *FoM*. For the design, we set *N* to 24, 32, and 40, respectively. Because we have four layers, each layer has *N* / 4 pixels and thus each pixel has a width of ${w}_{p}=\frac{4{\Lambda }}{{N}_{ }}$. We selected a thickness of 20 nm for each layer to ensure a low optical loss and film uniformity. To simulate the optical isolation function, an x-polarized plane wave was incident on the pixelated metamaterial structure. A rigorous coupled-wave analysis (RCWA) [37] combined with the enhanced transmittance matrix approach [38] and Li’s factorization rules [39] was used to calculate the electromagnetic field distribution for the cases ${\varvec{P}}_{1\to 2}$ and ${\varvec{P}}_{2\to 1}$ to obtain ${T}_{F}$ and ${T}_{B}$, respectively.

### QA-aided optimization of optical diodes

The QA-enhanced active learning scheme is iterative (see Fig. 2B), where each iteration has four steps: (1) Using the dataset consisting of binary vectors and the associated *FoMs* {***x***, *FoM*}, an FM surrogate model is trained, and its parameters are used to formulate a QUBO matrix for QA optimization. (2) The QA hardware (i.e., superconducting qubits in D-Wave Systems Inc.) accepts the QUBO matrix and samples the optimal state (supposed to be the ground state) of the QUBO model via adiabatic annealing. (3) RCWA evaluates the QA-identified optimal structure and calculates the associated *FoM*. (4) The dataset is updated by adding the QA-identified optimal structure and its RCWA-calculated *FoM* to {***x***:*FoM*}. This is used to retrain the FM model in the next iteration. The iteration is stopped after the optimal *FoM* identified remains unchanged for 90% of the last one hundred optimization cycles. Supplementary Notes I and II provide further details on the optimization process. It should be noted that the QA scheme can efficiently evaluate a given surrogate even when the optimization space is large (see Supplementary Note I). Hence, the QA scheme can identify an identical optimal design orders-of-magnitude faster than exhaustive or random searches (see Supplementary Note III). Furthermore, the QA scheme can be much more efficient compared to other conventional optimization schemes, which may require considerable time to evaluate surrogate models, especially when the optimization space is large. The RCWA calculation for a binary vector required 42 s on a 32-core AMD Ryzen Threadripper PRO 3975WX workstation. Hence, evaluating all the feasible configurations through exhaustive enumeration is impractically time-consuming.

**Fig. 3:**
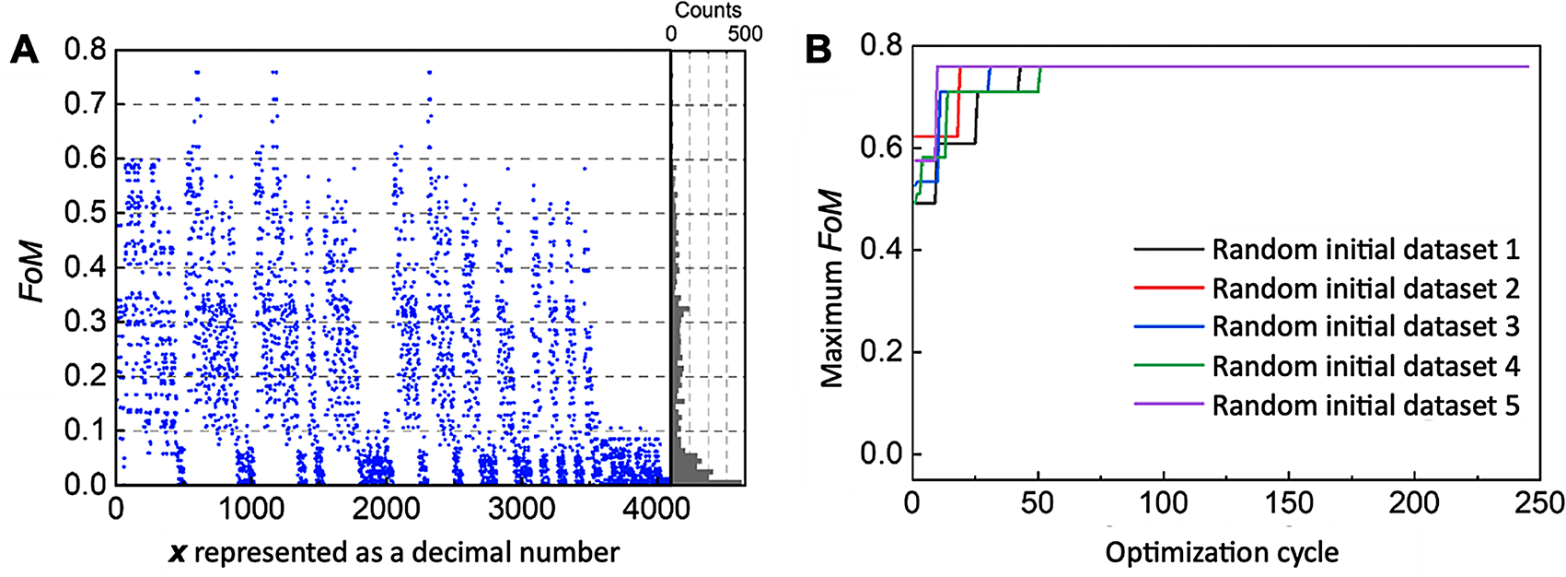
Benchmark study of QA-enhanced active learning scheme (**A**) Exhaustive enumeration result of *FoM* for the case of *N* = 12 for ${{\uplambda }}_{0}$ = 600 nm. RCWA evaluates all the 4,096 possible configurations. *x* is represented as a decimal number. The inset is the count-of-*FoM* distribution. (**B**) Maximum *FoM* as a function of optimization cycles with the QA-enhanced active learning scheme. The optimization process starts with five different random initial datasets

To validate the QA-enhanced active learning scheme, we first studied a candidate structure with *N* = 12, which we can afford to calculate the *FoMs* for all possible configurations exhaustively using RCWA to identify the global optimal state. For *N* = 12, there are 2^12^ (4,096) possible configurations that can be evaluated using RCWA in 172,032 s (approximately 48 h). Out of these configurations, the global optimal structure has an *FoM* of 0.7588 (Fig. 3A). Most structures have *FoM* values between 0 and 0.1, and the probability of identifying structures with a high *FoM* (> 0.75) is less than 0.1465% (see the *FoM* histogram in Fig. 3A). The iterative algorithm can determine the global optimal structure within 51 iterations (consuming at most 2,295 s) depending on the initial dataset (Fig. 3B). We note that FM training and QA consumed only a few seconds in each iteration. Therefore, the RCWA calculation consumed most of the time (2,142 out of 2,295 s) in the optimization process. The identified optimal *FoM* is 0.7588, which matches that obtained through the exhaustive search. This validation confirms that the QA scheme can successfully locate the global optimal state for *N* = 12, indicating its efficiency for larger *N* cases. The advantage of the QA scheme is expected to grow as the degree of freedom (*N*) increases.

**Fig. 4:**
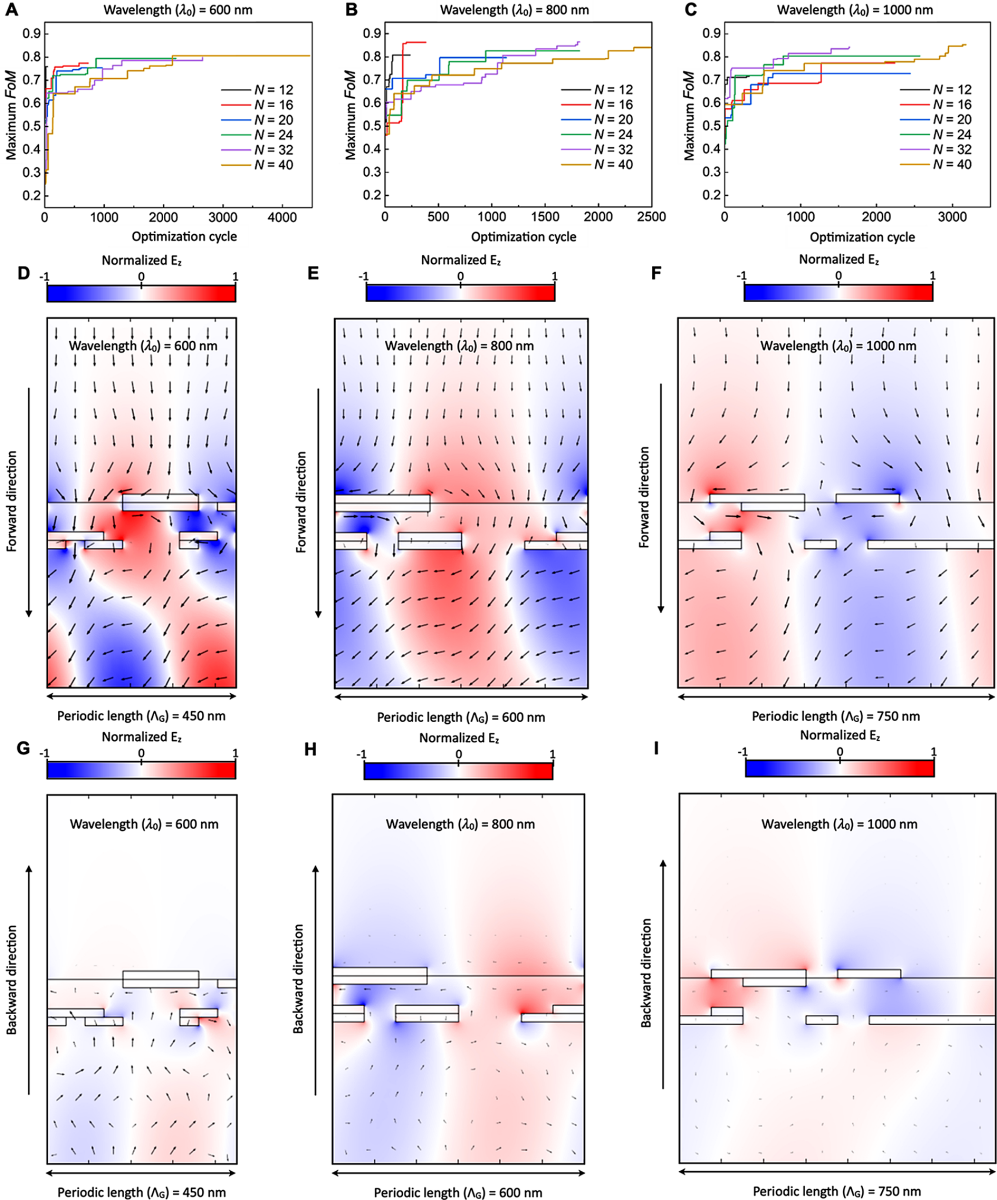
Optimized thin-film optical diodes at various target wavelengths (**A** to **C**) Maximum *FoM* as a function of optimization cycles with *N* = 12, 16, 20, 24, 32, and 40 for target wavelengths of (**A**) ${{\uplambda }}_{0}$ = 600 nm (${{\Lambda }}_{G}$ = 450 nm), (B) ${{\uplambda }}_{0}$ = 800 nm (${{\Lambda }}_{G}$ = 600 nm), and (**C**) ${{\uplambda }}_{0}$ = 1000 nm (${{\Lambda }}_{G}$ = 750 nm). (**D** to **I**) Electromagnetic field profiles for (**D**, **E**, **F**) forward cases and (**G**, **H**, **I**) backward cases of the optimal optical diodes at (**D**, **G**) ${{\uplambda }}_{0}$ = 600 nm (${{\Lambda }}_{G}$ = 450 nm), (**E**, **H**) ${{\uplambda }}_{0}$ = 800 nm (${{\Lambda }}_{G}$ = 600 nm), and (**F**, **I**) ${{\uplambda }}_{0}$ = 1,000 nm (${{\Lambda }}_{G}$ = 750 nm). The black arrow indicates the Poynting vector at the given spatial point. The length of the arrow is on a logarithmic scale. Normalized ${\text{E}}_{z}$ depicts the z-component of the electric field, normalized by its maximum. The solid black lines indicate the material boundaries

We then increase *N* to 16, 20, 24, 32, and 40 and target three wavelengths of 600 nm (${{\Lambda }}_{G}$ = 450 nm), 800 nm (${{\Lambda }}_{G}$ = 600 nm), and 1000 nm (${{\Lambda }}_{G}$ = 750 nm) to demonstrate the versatility of the QA-enhanced active learning scheme. For the largest *N* ( = 40), the optimal structures can be identified within a few thousand optimization cycles (see Fig. 4A , B and C), where up to 4,458 RCWA calculations are required for the optimization (${{\uplambda }}_{0}$ = 600 nm, see Fig. 4A). This is a very small fraction (4458/2^40^ ≈ 4 × 10^− 9^) of the whole optimization space. This efficient optimization is attributed to the acceleration of evaluating a given surrogate model by employing QA. The best *FoM*s of the identified optical diodes are 0.8064 for ${{\uplambda }}_{0}$ = 600 nm, 0.8648 for ${{\uplambda }}_{0}$ = 800 nm, and 0.8533 for ${{\uplambda }}_{0}$ = 1000 nm. This is comparable to the state-of-the-art macroscopic [40–44] and thin-film optical isolators [8–15]. In general, a higher *N* yields a higher *FoM*. However, it is noted that the best *FoM* is obtained when *N* = 32 for the wavelength of 800 nm. This discrepancy can be attributed to the variations in pixel size, as indicated by the pixel width. However, a structure having two times the number of pixels as another should exhibit a higher *FoM* or at least an equivalent one. This is because when one pixel is divided into two, the resultant structure gains an increased degree of freedom and resolution for optimization. Indeed, upon analysis, structures comprising 24, 32, and 40 pixels yield higher *FoM*s than those with 12, 16, and 20 pixels (Fig. 4A, B and C). It is worth noting that the active learning scheme can design optical diodes for other target wavelengths with the condition of ($\frac{{2\text{n}}_{1}\pi }{\lambda } <\frac{2\pi }{{{\Lambda }}_{G}}<\frac{2{\text{n}}_{2}\pi }{\lambda }$). The selection of periodic length determines the wavelength range that the optical isolation effect appears, with a peak achieved near the target wavelength (see Supplementary Note IV and Note V).

The identified optimal structures are counterintuitive and differ from those presented in previous studies [8–15], particularly in terms of their geometrical configurations (see Fig. 4D–I). The optimal binary vectors are 0000111100 0000111101 1,110,000,110 1,011,000,100 (*N* = 40) for ${{\uplambda }}_{0}$ = 600 nm, 11,100,000 11,100,000 10,110,001 10,110,011 (*N* = 32) for ${{\uplambda }}_{0}$ = 800 nm, and 0111011000 0011000000 0100000000 1,100,101,111 (*N* = 40) for ${{\uplambda }}_{0}$ = 1000 nm. These vectors are represented as pixelated metamaterial structures (from top left to bottom right). We note that the performance of the optical diode is sensitive to the structural design, which means optimization is important to achieve high *FoM*. For example, a change of geometry (adding one metal pixel to the optimal structure; 11100001 11,100,000 10,110,001 10,110,011 for ${{\uplambda }}_{0}$ = 800 nm) can lead to a great decrease in *FoM* from 0.8648 to 0.3028 (${T}_{F}:$ 0.8665 $\to$ 0.4651, ${T}_{B}:$0.0017 $\to$ 0.1623). To explore the mechanism of optical diodes, we estimated the intensities of the zeroth-, first-, and second-order diffracted light (see Table 1). It is noteworthy that the zeroth-order diffracted light is almost attenuated (transmissivity of 0.4–2%), which is the key to achieving a high-performance thin-film optical diode. However, for the forward case, the transmissivities of the first-order diffracted light (plus and minus orders) are not zero, and they contribute to a high value of ${T}_{F}$ (80–85%). It is also observed that the intensity of the positive first-order diffracted light is not equal to that of the negative light. This could be owing to the asymmetrical structure of the optical diode interface. The optical responses of the unit cell (not a single pixel), which includes metal pixels surrounded by dielectric pixels, are useful to investigate the mechanism of the optical diode effect. Hence, we further investigated the reflectivity and absorptivity. The results demonstrate that it is the reflection that mainly contributes to the optical diode effect for the diodes with the target wavelengths of 800 and 1000 nm while absorption mainly contributes to the effect for the diode with the target wavelength of 600 nm (see Table 1 and Supplementary Note IV).

**Table 1:** Optical characteristics of diffracted lights on optimized thin-film optical diode interfaces

	Wavelength	600 nm		800 nm		1000 nm	
	Diffraction Order	Forward (${\varvec{P}}_{1\to 2})$	Backward (${\varvec{P}}_{2\to 1})$	Forward (${\varvec{P}}_{1\to 2})$	Backward (${\varvec{P}}_{2\to 1})$	Forward (${\varvec{P}}_{1\to 2})$	Backward (${\varvec{P}}_{2\to 1})$
Transmissivity	1	0.2182	0	0.1567	0	0.2355	0
	0	0.0042	0.0042	0.0017	0.0017	0.0205	0.0205
	-1	0.5882	0	0.7081	0	0.6178	0
Reflectivity	1	0	0.2259	0	0.1941	0	0.0537
	0	0.0039	0.0431	0.0280	0.6626	0.0060	0.8375
	-1	0	0.1264	0	0.0225	0	0.0084
Absorptivity		0.1855	0.6004	0.1055	0.1191	0.1202	0.0799

We investigated the time-averaged Poynting vector profiles to visualize the optical isolation function for each wavelength (Fig. 4D–I). It is evident that the Poynting vectors smoothly pass the pixelated metamaterial structure through the dielectric pixels between the metal pixels when incident from medium 1 (i.e., the forward direction). Notably, the Poynting vector after the structure propagates in the off-normal direction owing to the asymmetric amplitudes of the first-order diffracted light. Meanwhile, when it is incident from medium 2 (i.e., the backward direction), the Poynting vector is almost blocked by the structure, rotated in the vicinity of the pixelated metamaterial structure, and is eventually reflected back into medium 2. To further study the optical properties of these structures, we investigated the z-components of the electric field profiles (see Fig. 4D–I). The field profiles show that for the forward case, intensive SPPs are coupled across each Ag pixel over the dielectric medium from the first to the fifth layers. This is similar to the SPP mode of the metal–dielectric grating. It shows in-phase wavefronts expanding toward medium 2 through the pixelated metamaterial structure, which is similar to previously reported optical thin-film diodes leveraging coupled SPPs [10, 12]. In contrast, the backward cases have weakened SPPs, which seem to be decoupled and localized at each metallic pixel, similar to the localized surface plasmons of nanoparticles.

### Experimental verification

**Fig. 5:**
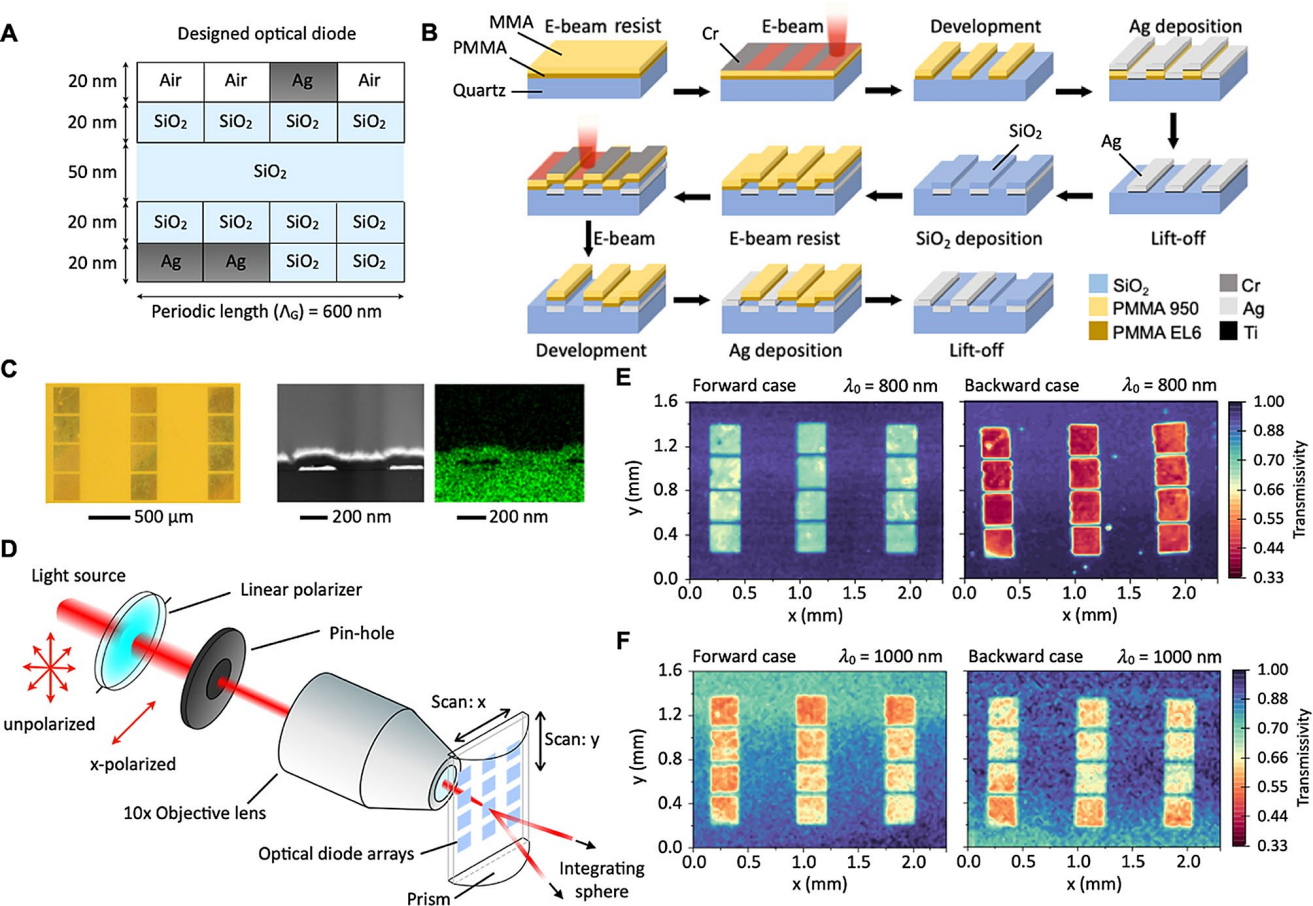
Experimental verification of the designed thin-film optical diode. (**A**) Selected optical diode with an *FoM* of 0.86 for the target wavelength of ${{\uplambda }}_{0}$ = 800 nm (${{\Lambda }}_{G}$ = 600 nm) at *N* = 16. (**B**) Schematic of the fabrication process of the selected optical diode with electron-beam (E-beam) lithography. (**C**) (Left) Optical microscope image of the thin-film optical diode 4 × 3 array on a quartz substrate. (Middle) Cross-sectional transmission electron microscope (TEM) image of the diode. (Right) Cross-sectional energy-dispersive X-ray analysis image of the cross-section for the TEM image. (**D**) Schematic of an experimental system for characterizing transmissivities of the fabricated optical diode. (E, F) Measured forward and backward transmissivities of the optical diodes array at (**E**) ${{\uplambda }}_{0}$ = 800 nm and (F) ${{\uplambda }}_{0}$ = 1000 nm

From the QA-designed optical diode structures for ${{\uplambda }}_{0}$ = 800 nm, we select one satisfying *FoM* > 0.8 and ${T}_{B}$ < 0.05, whose structure is not too complicated to fabricate using electron-beam (e-beam) lithography. The fabrication of the best diodes from the QA design requires up to four times of e-beam lithography, which involves expensive and time-consuming processes, potentially leading to many experimental challenges and imperfections. However, the selected structure needs only two times of e-beam lithography in its fabrication (binary vector is 0010 0000 0000 1100, *N* = 16). Hence, we can minimize the complexity of the fabrication while still using it as a case to test our QA design. As shown in Fig. 5A, the patterns of the metallic and dielectric pixels are simple compared with the optimal structures shown in Fig. 4D, E and F. Nevertheless, the theoretical estimation indicates that this optical diode still has a potential performance (*FoM* = 0.8632) with ${T}_{F}$= 0.8877 and ${T}_{B}$ = 0.0245 for the normally incident light with ${{\uplambda }}_{0}$ = 800 nm. This result implies that choosing an optical diode with relatively simple geometry in the pool of good candidates can be a promising strategy to relax fabrication complexity.

The optical diode is polarization sensitive. Thus, asymmetric transmission can be observed with the TM-polarized light (the electric field perpendicular to the stratified grating). The optical diode clearly shows asymmetric transmissivity for the TM polarized light in the wavelength range of 750 to 850 nm, although it is designed for the target wavelength of 800 nm. It is noted that the TE-polarized light (the electric field parallel to the stratified grating) is hard to couple to the SPPs of the stratified grating since the electric field oscillates along the direction of metal/dielectric interfaces [10, 11]. Hence, ${T}_{B}$ is high, and the difference between ${T}_{F}$ and ${T}_{B}$ is not large for the TE-polarized light (see Supplementary Note V).

E-beam lithography was used to fabricate the optical diode (see Fig. 5B and Method for details). The area of the optical diode was 270 μm × 270 μm, and we prepared a 4-by-3 array of the optical diode on the quartz substrate (Fig. 5C). We used customized spectroscopy combined with an optical microscope and motorized x-y stages to precisely control the position of the beam-exposed spot on the substrate (see Fig. 5D and Methods for details). A half-cylindrical prism was used in the forward case to capture the diffracted transmitted light using an optical integrating sphere.

The spectroscopy scans the 4-by-3 array of the optical diode interfaces and measures ${T}_{F}$ and ${T}_{B}$ as a function of position (Fig. 5E and F). This clearly shows the optical diode effects where the highest ${T}_{F}$ is ∽ 0.7963 and the lowest ${T}_{B}$ is ∽ 0.3821 which is in contrast to the non-structured flat interface (Fig. 5E). Additionally, the fabricated optical diode has wavelength selectivity, where the beam with a wavelength of 1000 nm cannot yield an obvious optical isolation function (Fig. 5F), This is because this case (${\uplambda }$ = 1000 nm and ${{\Lambda }}_{G}$ = 600 nm) does not satisfy $\frac{2{n}_{1}\pi }{{{\uplambda }}_{0}}<\frac{2\pi }{{{\Lambda }}_{G}}< \frac{2{n}_{2}\pi }{{{\uplambda }}_{0}}$. The observation agrees well with the RCWA-estimated spectral window of the optical isolation function (λ = 600 to 870 nm, see Supplementary Note V). However, the measured performance of the optical diode did not completely agree with the performance calculated from the RCWA. The measured ${T}_{B}$ is higher than that of the estimated values. We attribute this discrepancy to the structural differences between the fabricated and theoretically designed pixelated metamaterial structures. For example, the cross-sectional image of the fabricated optical diode interface shows bumps at the top SiO_2_ interface because of the buried silver metallic pixels (Fig. 5C). We used the finite element method to examine the effect of the bumps on the designed pixelated thin-film metamaterials. It was observed that the transmissivities of both forward and backward cases can be influenced. In addition, a Ti layer is needed as an adhesion layer between Ag and SiO_2_. However, the adhesion layer can affect the optical properties by increasing absorptivity, which causes a lowered ${T}_{F}$. According to the FEM simulations, the Ti adhesion layer causes the absorptivity to increase from 0.12 to 0.31, while maintaining low reflectivity (∽0), resulting in decreased transmissivity from 0.88 to 0.68 for the forward case. The measured transmission spectra differ from the simulation results, likely due to experimental deficiencies, such as the presence of bumps, the Ti layer, and the peeled-off top Ag patterns (see Supplementary Note VI). Despite these deficiencies, the optical diode still presents a good optical diode effect with a transmissivity contrast depending on the direction of light incident at a target wavelength We believe that enhancing the efficiency of the optical diodes can be achieved by including practical fabrication issues, such as the bump or Ti layer, within the active learning scheme. For instance, a model structure of an optical diode includes the bump and Ti layer, or the *FoM* definition includes the quantified fabrication complexity arising from these factors. This approach holds the potential to realize high-performance optical diodes by minimizing experimental errors and fabrication complexities. It is noted that unlike electrical diodes, which have a threshold voltage determined by the energy band alignment at a p-n junction, the optical diode does not have such a threshold [45]. However, they share similarities with thermal diodes in terms of the concept of asymmetric transmission of the energy flow of interest [2, 3].

## Conclusion

In conclusion, we designed thin-film optical diodes with a pixelated metamaterial structure at three specific wavelengths of ${{\lambda }}_{0}$ = 600, 800, and 1000 nm using the QA-enhanced active learning scheme. The designed diodes have counterintuitive geometrical configurations of metal–dielectric pixels but exhibit high *FoM*s of 0.80–0.85. The electromagnetic field profiles indicate that enhancing the coupling strength of the SPPs for the forward case and attenuating it for the backward case can be key factors in enabling the optical isolation function. Despite experimental deficiencies, the fabricated diode shows a forward transmissivity (0.7963) that is higher than the backward transmissivity (0.3821). Because of the limitations of the available qubits in the QA backend, the binary bit length is capped at ∽ 120 bits when a fully connected topology is used. To circumvent this limitation, the recommendation is to break down the problem into smaller parts by decomposing the QUBO matrix into sub-QUBOs [46]. This approach could potentially enable the application to handle finer pixels while adhering to the hardware constraints We also note that within the condition of ($\frac{{2\text{n}}_{1}\pi }{\lambda } <\frac{2\pi }{{{\Lambda }}_{G}}<\frac{2{\text{n}}_{2}\pi }{\lambda }$), varying other factors (e.g., the thickness of the Ag pattern, and the number of layers) can lead to different optimized *FoMs* as well as bandwidth. The area of ultrathin optical diode can be potentially scaled up using deep ultraviolet photolithography, potentially allowing it to be integrated into photonic applications such as photonic integrated circuits and photonic neural networks [47].

### Electronic supplementary material

Below is the link to the electronic supplementary material.

Supplementary Material 1

## Data Availability

The data that support the findings of this study are available from the corresponding author upon reasonable request.
