# Supplementary material for: Quantum annealing-aided design of an ultrathin-metamaterial optical diode
Source: Nano Converg. 2024 May 9;11:16. doi: 10.1186/s40580-024-00425-6 (PMC11082120; doi:10.1186/s40580-024-00425-6)
Supplement: Supplementary file 1 — Supplementary Material 1 [file 40580_2024_425_MOESM1_ESM.docx]

Supplementary Information

Quantum Annealing-aided Design of an Ultrathin-Metamaterial Optical Diode

Seongmin Kim^1,4,+^, Su-Jin Park^2,+^, Seunghyun Moon^1^, Qiushi Zhang^1^, Sanghyo Hwang^3^,

Sun-Kyung Kim^2,*^, Tengfei Luo^1,*^, Eungkyu Lee^3,*^

^1^Department of Aerospace and Mechanical Engineering, University of Notre Dame; Notre Dame, Indiana, 46556, United States.

^2^Department of Applied Physics, Kyung Hee University; Yongin-Si, Gyonggi-do, 17104, Republic of Korea.

^3^Department of Electronic Engineering, Kyung Hee University; Yongin-Si, Gyonggi-do, 17104, Republic of Korea.

^4^National Center for Computational Sciences, Oak Ridge National Laboratory, Oak Ridge, Tennessee 37830, United States.

*Corresponding authors: Sun-Kyung Kim, Tengfei Luo, and Eungkyu Lee

E-mail: Sun-Kyung Kim (sunkim@khu.ac.kr), Tengfei Luo (tluo@nd.edu), and Eungkyu Lee (eleest@khu.ac.kr)

**Supplementary Note I. Defining an objective function for the quantum annealing (QA) using the factorization machine (FM).**

We used xLearn, a high-performance open package for the FM, as a machine learning model. We developed MATLAB scripts to call the xLearn for training the FM model using the training datasets. Finding the optimal binary vector by evaluating the FM model in classical computers can take a significant time as the number of pixels increases due to the exponentially increasing number of total possible configurations (Fig. S1A). In particular, classical computers cannot conduct this prediction task for designing more than 27-pixeled metamaterial structures (Fig. S1B). Hence, we used the D-Wave QA (Advantage 4.1) to overcome the limitations of the classical computer. The D-Wave QA solver has a strong ability to find the ground state when the problem is formulated as a quadratic unconstrained binary optimization (QUBO) Hamiltonian as shown in the following equation (S1).

|  | $y=\sum_{i} Q_{i,i}x_{i}+\sum_{i<j} Q_{i,j}x_{i}x_{j}$ | (S1) |
| --- | --- | --- |

where $Q_{i,i}$ is a coefficient for linear terms, $Q_{i,j}$ for quadratic terms, and $x_{i}$ is a binary variable. Also, equation (S1) can be simply defined as equation (S2).

|  | $y=\sum_{x\in\left\{ 0,1 \right\}^{n}} \boldsymbol{x}^{T}Q\boldsymbol{x}$ | (S2) |
| --- | --- | --- |

With the dataset {***x***, figure-of-merit (*FoM*)}, FM is trained to provide an objective function that describes the relationship between the input ($x_{i}$) and *FoM* ($y$), which is given by equation (S3)

|  | $y:=w_{0}+\sum_{i=1}^{n} w_{i}x_{i}+\frac{1}{2}\sum_{f=1}^{k} \left[ \left( \sum_{i=1}^{n} v_{i,f}x_{i} \right)^{2}-\sum_{i=1}^{n} v_{i,f}^{2}x_{i}^{2}] \right]$ | (S3) |
| --- | --- | --- |

where model parameters $w_{0}$, $w_{i}$ and $v_{i,f}$ are bias, strength and interactions, respectively. The primary purpose is to find the minimum output y and the corresponding $\boldsymbol{x}$ using the FM model parameters. To mount this optimization problem on the QA domain, we express the FM model as the QUBO Hamiltonian. Since $x_{i}$ is a binary variable, the FM model (equation (S3)) can be expressed as equation (S4) [1].

|  | $y=b+\sum_{i} \boldsymbol{x}^{T}W_{i,i}\boldsymbol{x}+\sum_{i<j} \boldsymbol{x}^{T}V_{i,j}V_{i,j}^{T}\boldsymbol{x}$ | (S4) |
| --- | --- | --- |

where $b$ is bias, $W_{i,i}$ is linear and $V_{i,j}$ is interaction terms. Once omitting bias, which does not affect the matrix dimensions, the FM model in equation (S4) can be expressed as the QUBO Hamiltonian as equations (S1) and (S2). Consequently, the QUBO originated from the FM model parameters is used as an objective function in the QA hardware to rapidly predict the optimal binary vectors corresponding to the ground state of the QUBO Hamiltonian [2, 3]. Note that the QA is specialized to find the minimum energy state, so we multiplied *FoM* in the dataset by -1 to convert the maximization problem in the design space into a minimization problem in the QA domain.

**
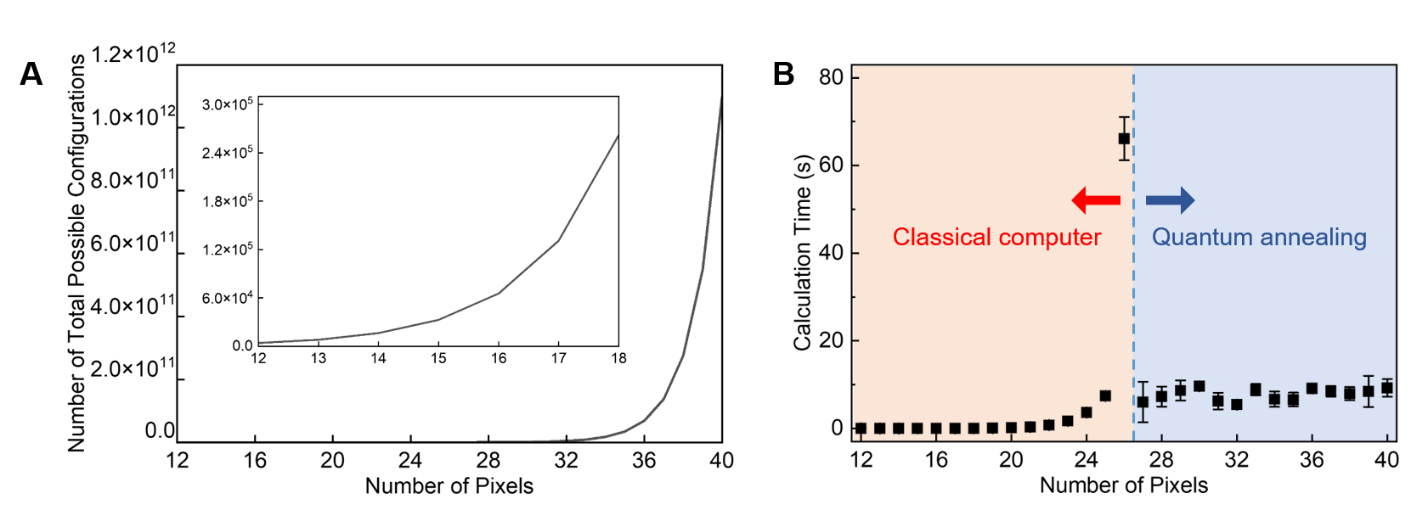
**

**Fig. S1.** (A) The number of total possible configurations as a function of the number of pixels. (B) Calculation time required to predict the optimal structure from the given objective function when using a classical or quantum annealer.

**Supplementary Note II. Details of the optimization process for identifying the optimal metamaterial structure for thin-film optical diode interfaces using the QA-enhanced active learning scheme.**

We use a *FoM*, which is the difference between forward and backward transmissivities (*i.e.*, *FoM* = $T_{F}-T_{B}$) as a metric of the optical diodes. The initial dataset has a data array including 25 randomly generated structures, and their corresponding *FoMs* are calculated by the rigorous coupled-wave analysis (RCWA). These 25 pairs of {***x***:*FoM*} are used as the initial training data for the FM. The QUBO is generated for implementing the objective function to the QA hardware from the trained FM model parameters (Supplementary Note I). Then, the optimal binary vector (***x*_po_**) that corresponds to the maximum *FoM****_po_*** can be rapidly predicted by the QA. Subsequently, RCWA evaluates the *FoM****_rcwa:po_*** of the ***x*_po_**, and the dataset is updated by adding the {***x*_po_**:*FoM****_rcwa:po_***} to the previous training dataset. We denote this one iteration as an optimization cycle. The QUBO is updated from an increasing amount of training data accumulated with the increasing number of optimization cycles. Hence, the scheme can successfully identify the optimal configuration in the design space despite the small amount of initial input data.

In the QA prediction (Fig. 2C), the binary vectors identified in previous cycles can be re-identified by the QA. In this case, a new random binary vector (***x*_random_**) is chosen from an unexplored area in the design space. Then, the *FoM* of ***x*_random_** is evaluated with the RCWA (*FoM****_rcwa:random_***), and a pair of {***x*_random_**:*FoM****_rcwa:random_***} is added to the dataset instead of {***x*_po_**:*FoM****_rcwa:po_***} for the next training. This is to diversify the sampling of the design space. Optimal configurations can be identified within dozens to hundreds of optimization cycles if the design space is small. However, the scheme can sometimes fail to find the global optimal configurations in the design space once getting stuck in local optima. Then, the scheme can hardly find any structures with high *FoMs* after a certain number of optimization cycles, suggesting the local optimum (Fig. S2A). When stuck in a local optimum, a new prediction in the QA-prediction step mostly repeats the previous prediction, thereby generating random vectors. Since most metamaterial structures have symmetric transmission, randomly generated vectors have mostly low *FoM* close to 0 (Fig. S2B). Thus, the iteration is hard to get out of the local optima in the design space. To resolve this issue, we do not use all data for the FM training when the number of data is over 200 (*i.e.*, n > 200). Our scheme uses randomly selected (n-200)/2+200 data for the FM training when n > 200, such that the local optimum problem can be resolved. Consequently, we can efficiently find optimal configurations, mitigating the local optimum problem, as shown in Fig. S2C.

We set some thresholds for stopping iterations to prevent excessive computational costs. We observed that the number of structures with $T_{F}$ greater than 0.8 until finding the optimal structure almost linearly increased with the increasing number of pixels. For $T_{F}$ > 0.8, there are 5 structures with *N* = 12, 14 structures with *N* = 16, 21 structures with *N* = 20, and 27 structures with *N* = 24. Hence, we can empirically estimate that there are 2**N*-18 structures with $T_{F}$ > 0.8 for the *N*-pixel diode. We terminate the iteration when the number of structures with $T_{F}$ > 0.8 is equal to 2**N*-18. In addition, the scheme generates random structures after getting stuck in local or global optima in the design space despite not using all data, which prevents finding better structures showing higher *FoM*. Therefore, we stop the iterations when 90% out of the last 100 data are already predicted structures. Consequently, the optimization continues until the scheme finds a certain number of metamaterial structures with high $T_{F}$ or gets stuck in local optima, without requiring the user’s intervention.

**
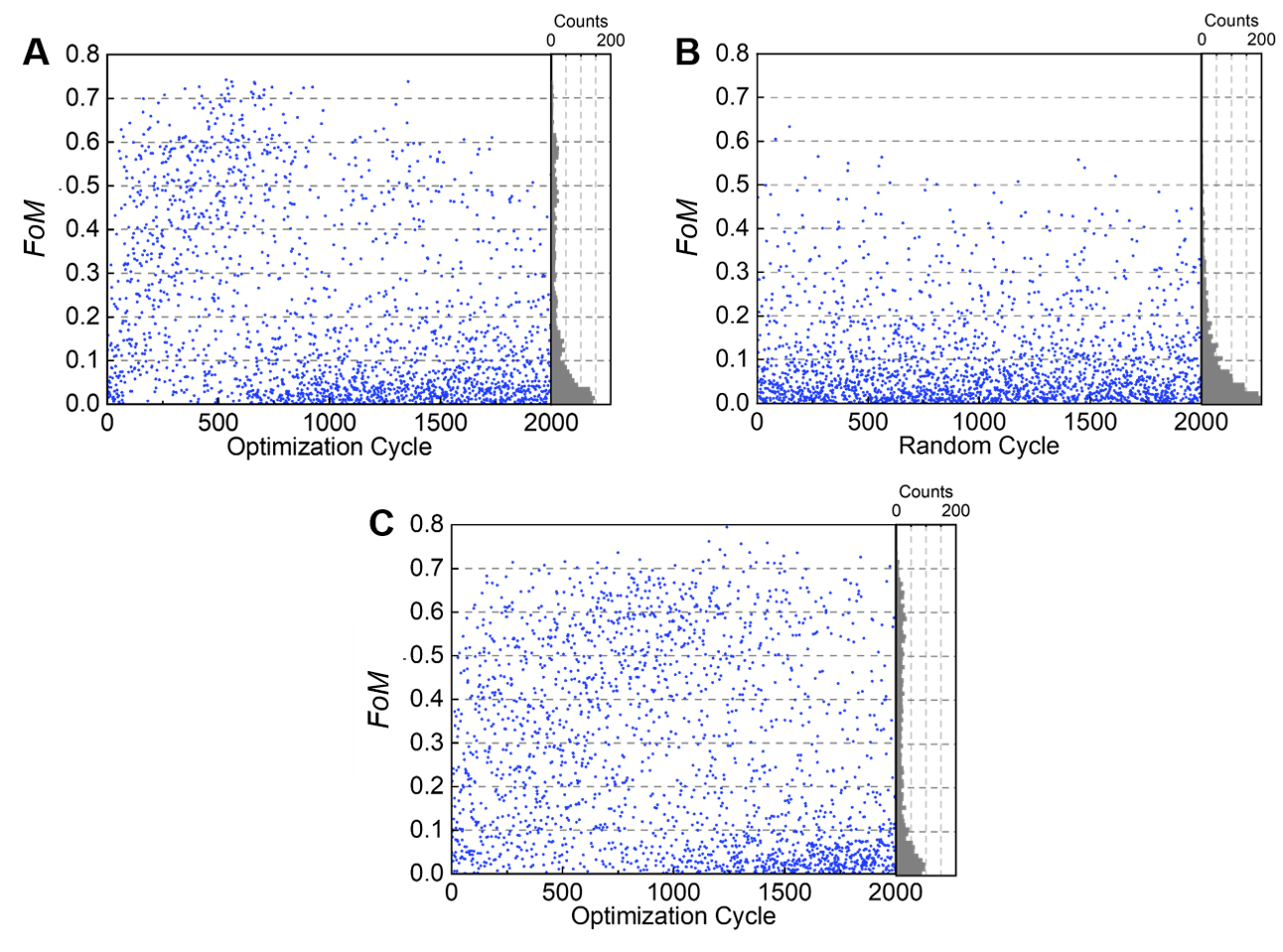
**

**Fig. S2.** Mitigation of the local optimum problem for the case of *N* = 24 ($\lambda_{0}$ = 600 nm). (A) *FoM_rcwa:po_* corresponding to *x*_po_ when using all data from the dataset for training the FM. (B) *FoM_rcwa:random_* corresponding to *x*_random_. (C*) FoM_rcwa:po_* corresponding to *x*_po_ when using a certain number of data from the dataset for training the FM.

**Supplementary Note III. Random optimization (RO).**

Without using an optimization scheme, *FoMs* can be evaluated with randomly generated structures using the RCWA. In this case, most of the structures have low *FoMs* (see Fig. S2B and Fig. 3A). Therefore, it typically requires considerable time to identify the optimal configuration. In a mathematical model (equation S5), the probability of identifying the optimal configuration by searching random structures within *n* cycles can be derived as:

|  | $\mathrm{probability} \left( \% \right)= \left( 1-\prod_{k=0}^{n-1} \frac{\left( n\left( S \right)- n\left( x \right)-k \right)}{\left( n\left( S \right)-k \right)} \right) \times100$ | (S5) |
| --- | --- | --- |

where $n\left( S \right)$ and $n(x)$ are the number of total candidates and the number of optimal configurations, respectively. Considering the mirror symmetry along the normal axis, $n(x)$ is set to (*N*/4) × 2. The optimal structures with *N* = 12 ($\lambda_{0}$ = 600 nm) are found within 51 optimization cycles when using the QA-enhanced active learning scheme (see Fig. 3B). However, the probability of identifying one of the optimal structures within 51 cycles by RO is only 7.25%. Optimal structures can be generally found over several hundreds of RO cycles, or may not be identified even with more than one thousand cycles (Fig. S3). RO requires 1,608 cycles to find one of the optimal configurations with 95% probability, which takes 67,536 seconds for RCWA calculations while the QA-enhanced active learning scheme requires only 1,395 seconds (31 optimization cycles) on average to find one of the optimal configurations. Furthermore, the probability of finding the optimal configuration rapidly decreases for designing higher-pixel optical diodes as $n\left( S \right)$ exponentially increases. For example, the design of a 40-pixel diode by RO is almost impossible with only a 0.0018% probability of finding the optimal structure even with 1 million cycles, which could take ~1.4 years. These results highlight the superiority of the QA scheme for the effective inverse design of functional materials.


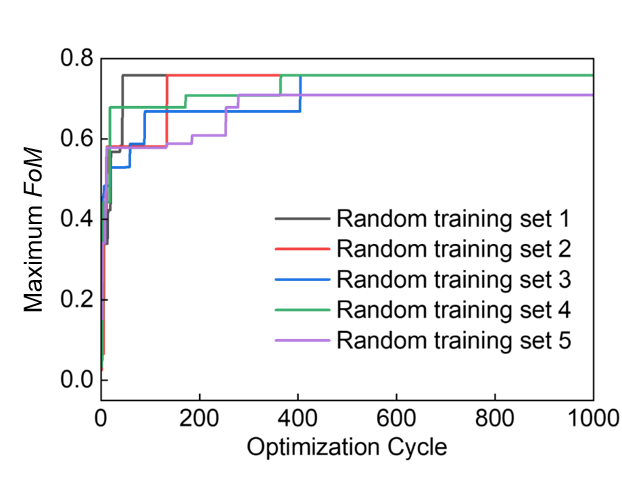


**Fig. S3**. Maximum *FoM* as a function of iteration when using random search started with five different random initial datasets (*N* = 12 for $\lambda_{0}$ = 600 nm).

**Supplementary Note IV. Analysis of reflectivity and absorptivity of the optical diode.**

We further calculate the reflectivity and absorptivity of the optimally designed optical diode (*N* = 32, $\lambda_{0}$ = 800 nm and $\Lambda_{G}$ = 600 nm) in a wavelength range of 450 to 1000 nm to investigate the origin of the asymmetric transmissivity. As can be seen in Fig. S4, the backward incident light is mostly reflected (*R_B_* > 0.85) and little absorbed (*A_B_* ~0.12) while the forward incident light is rarely reflected (*R_F_* ~0.03) and little absorbed (*A_F_* ~0.11) near the target wavelength (λ = 800 nm), which results in a high *T_F_* and low *T_B_* (Table 1).


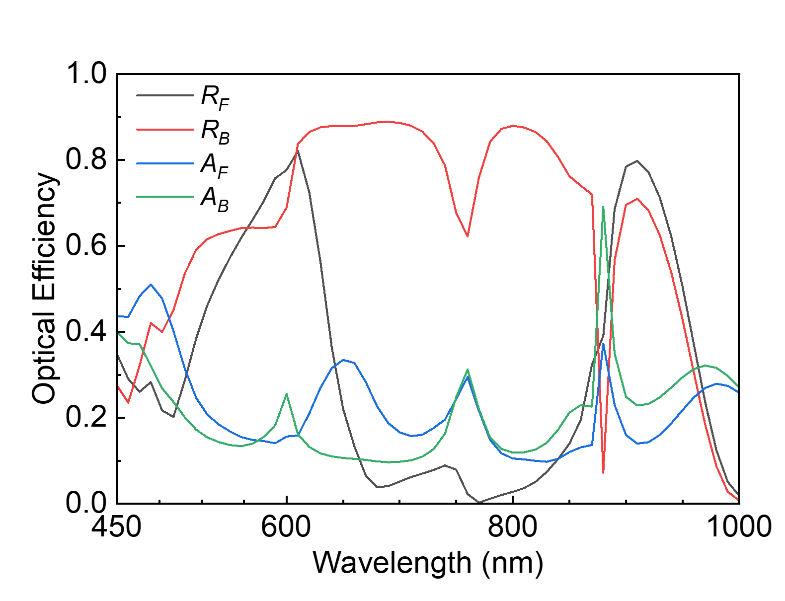


**Fig. S4.** RCWA-estimated reflectivity (*R_F_* and *R_B_*) and absorptivity (*A_F_* and *A_B_*) for the forward and backward light at wavelength λ = 450 to 1000 nm.

**Supplementary Note V. RCWA-estimated spectral window of the optical isolation effect.**

Transmissivities of the fabricated optical diode (*N* = 16, $\lambda_{0}$ = 800 nm and $\Lambda_{G}$ = 600 nm) for forward and backward cases are calculated for λ = 450 to 1000 nm using RCWA. The designed optical diode is polarization-dependent, so it presents optical diode effects with the TM-polarized light but not the TE-polarized light. To verify the polarization dependency of the optical diode, we calculated the forward and backward transmissivities of the diode (***x*** = 0100 0000 0000 1100) with the TM and TE-polarized lights. Fig. S5 demonstrates the optical diode is polarization dependent, showing a great optical diode effect for the TM-polarized light at the target wavelength ($\lambda_{0}$ = 800 nm).


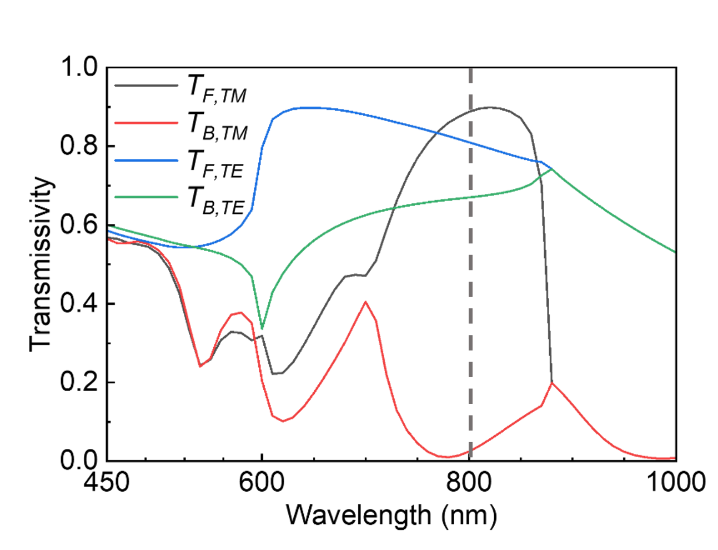


**Fig. S5.** Investigation of the polarization dependency of the optical diode. Forward and backward transmissivities of the ideally structured optical diode (*N* = 16, $\lambda_{0}$ = 800 nm and $\Lambda_{G}$ = 600 nm) are calculated with the TM- (*T_F,TM_* and *T_B,TM_*) and TE- (*T_F,TE_* and *T_B,TE_*) polarized lights.

Fabrication processes can pose experimental deficiencies, making bumps on the surface and requiring an adhesion layer for metal depositions on dielectric materials. Assuming the bumps with trapezoidal shapes, a model (Fig. S5A) is used for RCWA calculation. Fig. S5B shows calculated transmissivity ($T_{F}$and $T_{B}$) of the optical diode. The optical diode shows the optical diode effect in a wavelength range of 600 to 870 nm, especially showing a great performance at the target wavelength (800 nm). In other wavelength regions (*i.e.*, λ < 600 nm, and λ > 870 nm), the diode does not show the optical isolation effect because it cannot meet the condition of $\frac{2n_{1}\pi}{\lambda_{0}}<\frac{2\pi}{\Lambda_{G}}< \frac{2n_{2}\pi}{\lambda_{0}}$, which makes $T_{F}$ and $T_{B}$ almost the same (Fig. S5B).


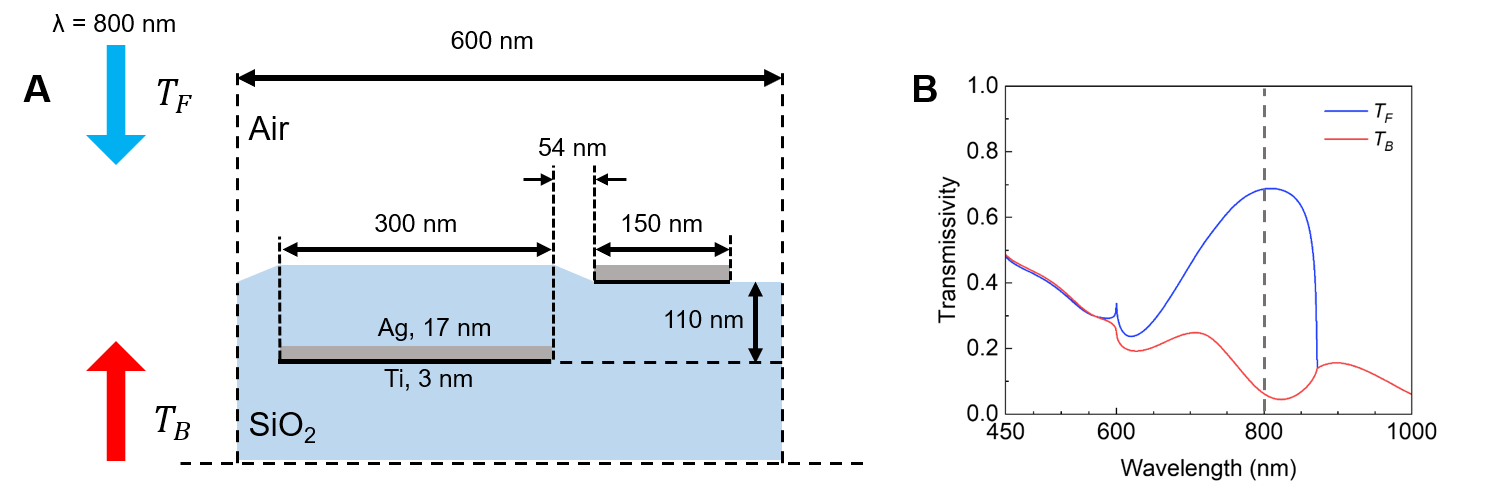


**Fig. S6.** Investigation of the optical isolation effect of the optical diode (*N* = 16, $\lambda_{0}$ = 800 nm and $\Lambda_{G}$ = 600 nm), which includes experimental imperfections. (A) Model for RCWA estimation. (B) RCWA-estimated transmissivity for the forward and backward cases in wavelength λ = 450 to 1000 nm.

**Supplementary Note VI. Analysis of transmissivities of the optical diode with experimental imperfections.**

The fabricated diode has a different shape from the ideal design since we did not flatten the deposited SiO_2_ layer after the fabrication of the metal pixels on the substrate, so the top layer has bumps with a ~20 nm thickness. In addition, the SiO_2_ bumps on the top may not have a sharp rectangular shape, instead, it may be a trapezoidal shape (see Fig. S6A). Furthermore, we have to deposit 3 nm of Ti on the SiO_2_ surface before depositing an Ag layer, making 20 nm of metal gratings, to enhance adhesion between Ag and SiO_2_. However, this thin Ti layer lowers transmissivity. To examine the effect of the SiO_2_ bumps and Ti adhesion layer, FEM simulations (COMSOL Multiphysics®) are conducted to calculate $T_{F}$ and $T_{B}$ of the selected optical diode (***x*** = 0010 0000 0000 1100, $\lambda_{0}$ = 800 nm and $\Lambda_{G}$ = 600 nm) with and without those imperfections. The model for the simulations is shown in Fig. S7. The bump affects transmissivities, increasing $T_{F}$ of 0.0106 and decreasing $T_{B}$ of 0.0069, while the Ti adhesion layer affects transmissivities more, decreasing $T_{F}$ of 0.0642 and $T_{B}$ of 0.0004. Hence, it is demonstrated that these experimental imperfections affect the transmissivities of the fabricated optical diode.


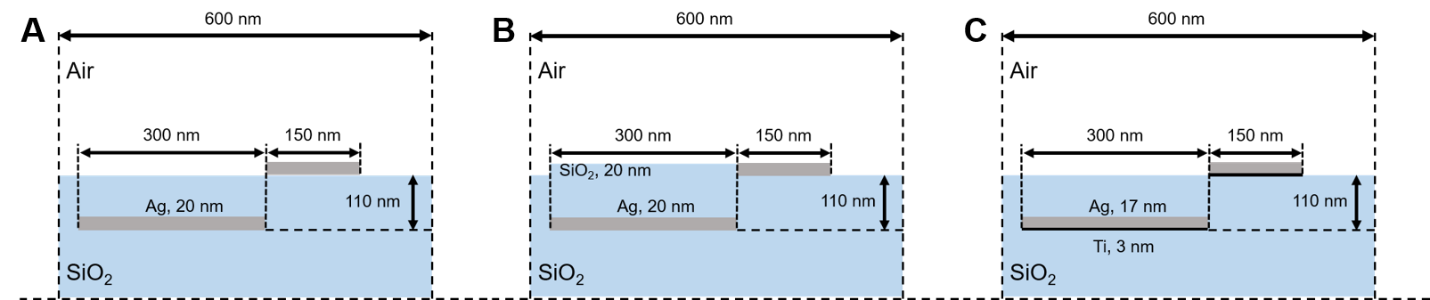
**Fig. S7.** Schematics of the optical diode to investigate experimental imperfections (*N* = 16, $\lambda_{0}$ = 800 nm and $\Lambda_{G}$ = 600 nm). (A) The optical diode without experimental imperfections. (B) The optical diode with the 20 nm of SiO_2_ bump on the surface. (C) The optical diode with 3 nm of adhesion (Ti) layer between Ag and SiO_2_.

We compare the measured transmissivity and RCWA-estimated transmissivity of the optical diode. When considering an optical diode with expected experimental imperfections and a peeled-off top Ag layer (Fig. S8A), the estimated transmissivities show similar amplitudes as those of the measured transmissivity within the wavelength range of 600 nm to 900 nm (Fig. S8B). However, the estimated transmissivities deviate from the measured transmissivities at the wavelength > 900 nm. These results suggest that the measured transmissivities arise from a combination of various imperfections present in the fabricated optical diodes. This is why the 800 nm results are better than the 1,000 nm results in experiments (Fig. 5E and 5F in the main text). We note that we are currently limited by the accuracy of the nanofabrication to fabricate exactly the same structure as designed in the QA scheme. We believe that with the advanced industrial processes, such as those using extreme ultraviolet lithography, the exact structures as designed by the QA scheme are realizable and the resulting performance will be similar to that predicted. Nevertheless, our experiments still show the proof-of-concept for using QA for optical metamaterial design and optimization.


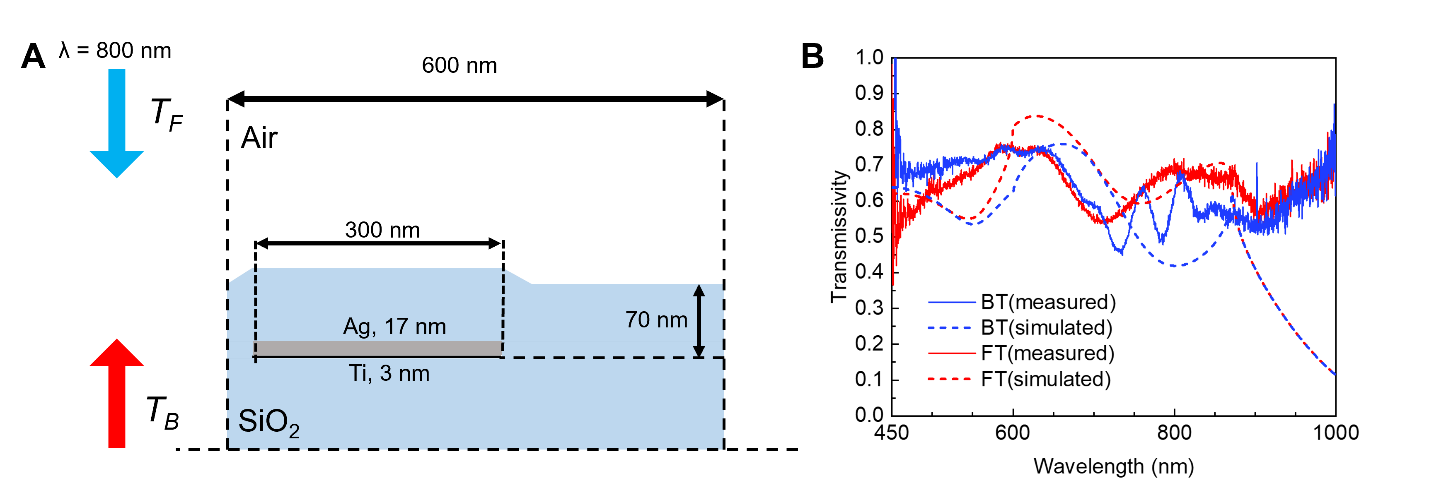


**Fig. S8.** (A) Schematic of the optical diode with a peeled-off top silver layer. (B) Measured and RCWA-estimated transmissivities for the forward and backward cases in the wavelength range of 450 to 1,000 nm.

Fig. S9 shows the diffracted light with the fabricated optical diode for forward and backward incident light. We observe light spots after passing the incident light through the optical diode or the glass sample (Fig. S9A). For the forward incident light, three spots are observed with the optical diode, indicating diffraction orders of -1, 0, and 1. On the other hand, only one spot corresponding to the 0^th^-order diffraction is observed with the glass sample (Fig. S9B). For backward incident light, only one spot representing the 0^th^-order diffraction is observed with both the diode and the glass sample (Fig. S9C). These results highlight that the optical diode functions to generate the 1^st^-order diffractions only for the forward incident light, enabling asymmetric transmission. Although the 0^th^-order diffraction is not perfectly attenuated due to experimental imperfections, the 1^st^-order diffractions observed only with the optical diode for the forward case demonstrate the optical diode performance.

**Fig. S9.** The far-field intensity measurement of diffracted light with the optical diode (*N* = 16, $\lambda_{0}$ = 800 nm and $\Lambda_{G}$ = 600 nm) and glass. (A) Schematic of the experiments to observe diffracted light. (B) Experimental results for the forward incident light. (C) Experimental results for the backward incident light.

**Supplementary references**

1. S. Rendle, in *In Proc. 2010 IEEE Int. Conf. Data Mining* (2010)

2. K. Kitai, J. Guo, S. Ju, S. Tanaka, K. Tsuda, J. Shiomi, R. Tamura, *Phys. Rev. Res.* **2**, 013319 (2020)

3. S. Kim, W. Shang, S. Moon, T. Pastega, E. Lee, T. Luo, *ACS Energy Lett.* **7**, 4134 (2022)
